# Basic magnesium sulfate@TiO_2_ composite for efficient adsorption and photocatalytic degradation of 4-dodecylmorpholine in brine

**DOI:** 10.1038/s41598-024-59921-8

**Published:** 2024-04-23

**Authors:** Zhongmei Song, Huifang Zhang, Liang Ma, Miao Lu, Chengyou Wu, Qingqing Liu, Xuefeng Yu, Haining Liu, Xiushen Ye, Zhen Ma, Zhijian Wu

**Affiliations:** 1 Key Laboratory of Green and High-end Utilization of Salt Lake Resources, Qinghai Institute of Salt Lakes, Chinese Academy of Sciences, Xining, 810008 China; 2 Qinghai University, Xining, 810016 China; 3 Qinghai Salt Lake Industry Co., Ltd., Golmud, 816000 China; 4 University of Chinese Academy of Sciences, Beijing, 100049 China

**Keywords:** Basic magnesium sulfate, Adsorption, Photocatalysis, 4-dodecylmorpholine, Degradation, Environmental sciences, Materials science

## Abstract

More than 70% of the potash fertilizer globally is produced by the froth flotation process, in which 4-dodecylmorpholine (DMP) serves as a reverse flotation agent. As the potash fertilizer production rapidly rises, the increased DMP levels in discharged brine pose a threat to the production of high-value chemicals. In this paper, composite particles of basic magnesium sulfate@TiO_2_ (BMS@TiO_2_) were prepared using a simple and mild loading method. These particles were utilized for the adsorption and photocatalytic degradation of DMP in brine. Compared with normal powdered materials, the granular BMS@TiO_2_ in this study can be easily separated from liquid, and the degradation intermediates will not enter the brine without causing secondary pollution. BMS@TiO_2_ consists of 5·1·7 phase (5Mg(OH)_2_·MgSO_4_·7H_2_O) whisker clusters embedding 2.3% TiO_2_. The adsorption equilibrium of DMP on BMS@TiO_2_ particles was achieved through hydrogen bonding and pore interception with the adsorption capacity of approximately 5 mg g^−1^ after 6 h. The photodegradation efficiency of DMP adsorbed on BMS@TiO_2_ reached about 92% within 16 h, which is compared with that of pure TiO_2_ nanoparticles. Additionally, excellent stability and recyclability of BMS@TiO_2_ were also observed in five cycle tests of adsorption and photocatalytic degradation of DMP, and the possible photocatalytic degradation pathways and mechanism of DMP are proposed following molecular electrostatic potential analysis. This work provides a sustainable and environmentally friendly approach for eliminating organic micropollutants from water environments.

## Introduction

Salt lakes contain large amounts of key minerals such as potassium and lithium, which are exploited for principally producing potash fertilizer, potassium nitrate for stored-heat molten salt, lithium carbonate in battery grade and so on. And these salt products are predominantly applied in agriculture and new energy, playing a vital role in promoting the economic and social development. Consequently, a series of steps are generally adopted to firstly extract potassium by flotation, and then lithium by membrane successively for sufficiently improving the comprehensive utilization efficiency of natural resources in salt lakes. Nevertheless, with the separation and enrich of potash resources forward, thousands of tons of flotation agents inevitably remain in potassium salt products, the effluent brine and the environment annually. In particular, the residues of flotation agent bring great challenges to the purification of products^1^, and simultaneously increase the risk of membrane fouling in the subsequent procedures for extract lithium^2^. 4-dodecylmorpholine (DMP) is commonly used as a flotation collector in the reverse froth flotation procedure^3^, and else assessed as secondary pollutant to ecological environment^4^. Therefore, the removal of DMP is necessary for further purification of related products and an important measure to protect the ecological environment.

The primary techniques for eliminating organic matter in wastewater include natural sedimentation^5^, coagulation^6^, biological treatment^7^, advanced oxidation^8^, and adsorption^9^. The study about the photocatalytic oxidation performance of titanium dioxide for the degradation of DMP and octadecylamine (ODA), confirms the feasibility of photocatalytic oxidation for the removal of flotation agents^10^. However, the catalyst, usually in the form of a powder, results in unsatisfactory reuse performance, thus restricting its further application. Adsorption method can effectively separate organic micropollutant from brine, in most cases, also requires desorption and regeneration procedures, whereas photocatalytic oxidation can achieve degradation of the adsorbed organic micropollutants and regenerate the adsorption material without additional chemical regenerants^11,12^. Therefore, the combination of the two methods is expected to enhance removal efficiency of organic micropollutants.

Adsorbent materials, such as resin^13^, metal-organic framework adsorbents^14^, activated carbon^15^, clay minerals^16–19^ and molecular sieves^20^ have been investigated extensively. Clay minerals, molecular sieves, and metal-organic frameworks are mostly in powder form, difficult to separate from liquids, while organic polymer resin may undergo aging by repeated illumination of ultraviolet light^21–24^. Activated carbon is often utilized as a carrier of photocatalysts for removing organic pollutants from wastewater. Liu et al. prepared an activated carbon photocatalyst supported by titanate nanotubes using the hydrothermal method to adsorb and photodegrade polycyclic aromatic hydrocarbons (PAHs). The maximum adsorption capacity was found to be 12.1 mg g^−1^, and PAHs could be photodegraded under ultraviolet light^25^. Zhu et al. developed an activated carbon composite supported by titanate nanotubes for removing perfluoro-2-propoxypropionic acid (GenX) with a photodegradation efficiency of 70%^26^. However, a long-term hydrothermal process^25–27^ or calcination^28,29^ is typically required for the deposition of the photocatalyst onto the adsorbent materials, in which the loading efficiency of the photocatalyst is uncontrollable^30–32^.

Cement-based materials are not only commonly used as building materials, but also can be doped with photocatalysts for photodegrading organic pollutants in water. Kumar et al. mixed BiVO_4_ photocatalyst into Portland cement paste and coated it on the prepared concrete spheres for oxidative degradation of methylene blue with the degradation efficiency of 58% in 240 min under visible light^33^. He et al. introduced a self-made photocatalyst into Portland cement for photocatalytic oxidation of methyl orange, and the degradation efficiency of methyl orange was approximately 50-65% under ultraviolet irradiation^34^. Zhou et al. spread the photocatalyst K-g-C_3_N_4_ powder onto the surface of uncured Portland cement and covered it with a glass slide for loading photocatalyst onto the cement surface^35^. The loading process of the photocatalyst on the above cement-based materials is mild and easy to operate. However, Portland cement is weakened and disintegrated by chemical reactions involving base exchange and the consequent leaching out of essential hydraulic components of the structure in salt solution^36^. Basic magnesium sulfate (BMS) possesses numerous distinct advantages, including high strength, light weight, good salt resistance, and hydrothermal resistance^37,38^. However, there is little publication available regarding BMS doped with photocatalysts for photodegrading DMP flotation agents in brine.

Herein, we have developed a facile approach to prepare BMS@TiO_2_ composite with controllable TiO_2_ loading and easy solid-liquid separation. In order to prevent possible degradation intermediates into brine, resulting in secondary pollution, BMS@TiO_2_ particles after adsorbing DMP were separated from solution, and then were directly irradiated by ultraviolet light for photodegrading DMP. Combined with textural and electrochemical characterization, a series of experiments were performed to investigate and elucidate the adsorption and photodegradation performance of BMS@TiO_2_ particles toward DMP in brine. Compared with commercial TiO_2_ powder, BMS@TiO_2_ particle only containing 2.3% TiO_2_ presents similar adsorption and photocatalytic degradation efficiency, as well as more efficient reusability. This work will provide new fabrication strategies for developing composite photocatalysts convenient for large-scale application in the future.

## Materials and methods

### Chemicals

Basic magnesium carbonate (Mg(OH)_2_·4MgCO_3_·xH_2_O) was purchased from Beijing Mairuida Technology Co., Ltd (Beijing, China). Magnesium sulfate (MgSO_4_·7H_2_O) was purchased from Tianjin Fengchuan Chemical Reagent Technology Co., Ltd (Tianjin, China). Citric acid monohydrate (C_6_H_8_O_7_) was purchased from Tianjin Hengxing Chemical Reagent Manufacturing Co., Ltd (Tianjin, China). Manganese dioxide (MnO_2_) was purchased from Tianjin Baishi Chemical Co., Ltd (Tianjin, China). Hydrogen peroxide (H_2_O_2_, 30%) was purchased from Tianjin Damao Chemical Reagent Factory (Tianjin, China). Methylorange (C_14_H_14_N_3_NaOS) was purchased from Beijing Reagent Factory (Beijing, China). Hydrochloric acid (HCl, 37%) was purchased from Sichuan Xi Long Chemical Co., Ltd (Sichuan, China). Sodium hydroxide (NaOH) was purchased from Xi’an Chemical Reagent Factory (Xi’an, China). 1,2-dichloroethane (1,2-ClCH_2_CH_2_Cl), glacial acetic acid (CH_3_COOH), anhydrous sodium acetate (CH_3_COONa), titanium dioxide (TiO_2_, anatase phase) and calcium stearate (C_36_H_70_CaO_4_) were purchased from Shanghai Mclean Biochemical Technology Co., Ltd (Shanghai, China). All chemicals were of analytical grade or higher. Besides, 4-dodecylmorpholine (C_16_H_33_NO) was industrial grade and provided by the Qinghai Salt Lake Industry Co., Ltd (Qinghai, China). Table S1 of the supplementary information (SI) presents the physicochemical properties of 4-dodecylmorpholine.

### Synthesis of BMS@TiO_2_

**Figure 1:**
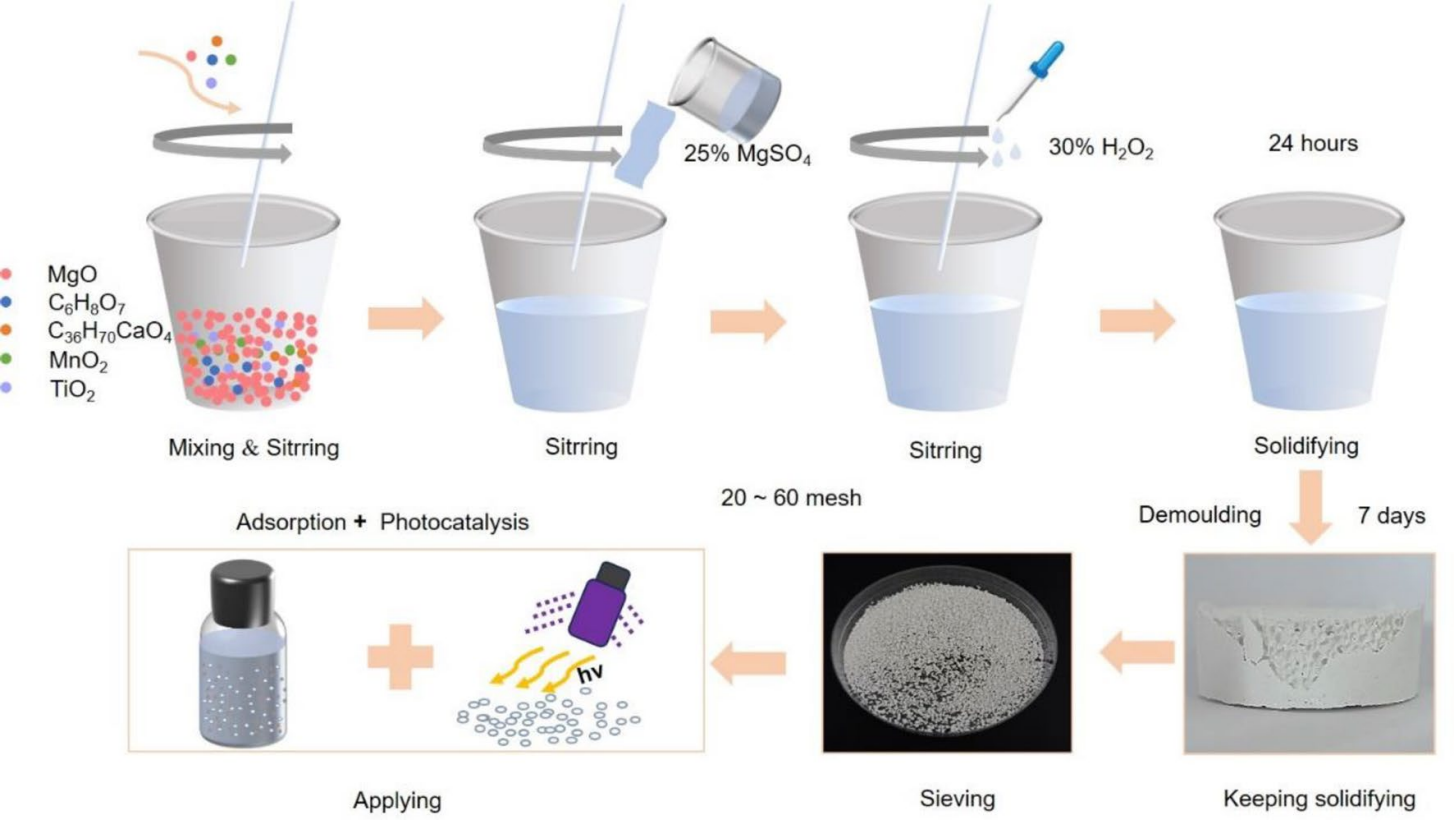
Synthesis schematic diagram of BMS@TiO_2_.

The synthesis method of BMS@TiO_2_ is shown in Fig. 1. Firstly, 5 g of laboratory-prepared active magnesium oxide from basic magnesium carbonate, 0.5 g of TiO_2_, 0.015 g of calcium stearate, 0.025 g of citric acid, and 0.002 g of MnO_2_ were mixed in a mold. Then, the mixture powder was combined with 16 g of a 25% magnesium sulfate solution and quickly whisked to form a uniform slurry. Finally, 1.5 g of a 30% H_2_O_2_ solution was added into the mixture slurry to induce foaming until the mixture was solidified. The solid sample obtained after demoulding and constant solidifying for 7 days at room temperature condition, was labeled as BMS@TiO_2_ and sieved to 20-60 mesh for adsorption and photocatalytic degradation experiments.

### Characterization

The surface morphology and elemental mapping of the samples were examined by scanning electron microscopy (SEM, SU8010, Hitachi, Japan) equipped with energy-dispersive X-ray spectroscopy (EDS). The microstructure was observed via transmission electron microscopy (TEM, F200, Jeol Ltd.). The crystal phases of the samples were analyzed using X-ray diffraction (XRD-6000, Shimadzu) with Cu-Kα irradiation. The porous characteristics and the specific surface area (S_BET_) were determined by the N_2_ adsorption/desorption method at a NOVA2200e instrument (Quantachrome). The electronic properties were measured through the electron paramagnetic resonance spectrometer (EPR, A300, Bruker, Germany). The UV-vis diffuse reflectance spectrometry (UV-DRS) analysis was performed on a Lambda750UV/VIS spectrophotometer. The chemical functional groups of prepared samples were analyzed by a Fourier-transform infrared spectrophotometer (FTIR, Bruker TENSOR37, USA). Total organic carbon (TOC) was recorded with a TOC analyzer (Analytikyena, C/N3100, Germany). The electrochemical measurements were performed on a Wuhan Coster electrochemical workstation (CS310H) with a conventional three-electrode cell.

### Photometric determination of DMP

Under a weak acidic environment, a 1:1 coordination reaction can occur between DMP and methyl orange. The product is bright yellow and soluble in 1,2-dichloroethane. When DMP remains in the lower organic phase and methyl orange enters the upper aqueous phase, the product will decompose under strong acidic conditions^39,40^. In the wavelength range of 450–550 nm, the acid methyl orange produced by decomposition exhibits a unique absorption peak. Quantitative measurement of DMP concentration can be done using a linear relationship between absorbance and DMP concentration (Fig. S1a). A calibration based on the Beer-Lambert law was used to quantify the concentrations of DMP. A detailed description of the analysis is provided in the supplementary materials, and the standard curve is shown in Fig. S1b.

### Adsorption experiment

The adsorption capacities of BMS@TiO_2_ for DMP were observed experimentally. Adsorption experiments were conducted by adding 0.1 g of BMS@TiO_2_ particles in 50 mL of DMP solution in a dark environment with variations in adsorption time (0, 0.5, 1, 1.5, 2, 3, 4, 5, 6, 8, 12, 16, 20, 24, and 32 h), operating temperature (25, 35, and 45 °C), initial concentration (15, 20, 25, 30, and 35 mg L^−1^), and co-existing salt (NaCl, KCl, and MgCl_2_). Concentrations of DMP before and after adsorption were determined by the UV-vis spectrophotometer, and the absorption capacity (*q*_*t*_, mg g^−1^) of DMP on materials and removal efficiency (*R*, %) were calculated via:

1 $$q_{t} = \frac{(C_{0} - C_{t})V}{m}$$

2 $$R = \frac{C_{0} - C_{e}}{{C_{e}}} \times 100\%$$

where *C*_*t*_ (mg g^−1^) is the concentration of DMP at time *t* (h), *C*_0_ and* C*_*e*_ (mg L^−1^) are the initial and equilibrium concentrations of DMP, respectively, *V* (L) is the total volume of the solution, and *m* (g) is the mass of adsorbent.

### Photocatalysis and reuse of BMS@TiO_2_

In order to prevent degradation intermediates into the solution, resulting in secondary pollution, BMS@TiO_2_ particles after adsorbing DMP were separated from solution, and then directly irradiated for photodegrading DMP by ultraviolet light source of 280-380 nm (9 cm away from the light source) in a photocatalytic glass reactor with a quartz cover. After photocatalytic degradation, the particles were washed by water and dried at 30 °C for a duration of 12 h for next adsorption experiment. The photocatalytic degradation rate (*η*) of DMP on BMS@TiO_2_ was measured by dissolving the particles at a certain photodegradation time with 15 mL of a 1 mol L^−1^ sulfuric acid solution, and calculated though the mathematical expression shown in formula (3). Pseudo-first-order and pseudo-second-order kinetic models were used to study the photocatalytic degradation behavior of DMP on BMS@TiO_2_, and the mathematical expressions are shown in formulas (4) and (5)^41,42^.

3 $$\eta = \frac{m_{0} - m_{t}}{{m_{0}}} \times 100\%$$

4 $${\text{ln}}\frac{m_{0}}{{m_{t}}}{ = }k_{1}t$$

5 $$\frac{1}{m_{t}} = \frac{1}{m_{0}}k_{2}t$$

where *m*_0_ and *m*_*t*_ represent the mass of DMP on the BMS@TiO_2_ at a certain photodegradation time (mg).* k*_1_ (h^−1^), and *k*_2_ (h mg^−1^) are pseudo-first-order and pseudo-second-order kinetic model constants, respectively, and *t* is the photodegradation time (h).

## Results and discussion

### Characterization of BMS@TiO_2_

#### Surface morphology and chemical composition of BMS@TiO_2_

**Figure 2:**
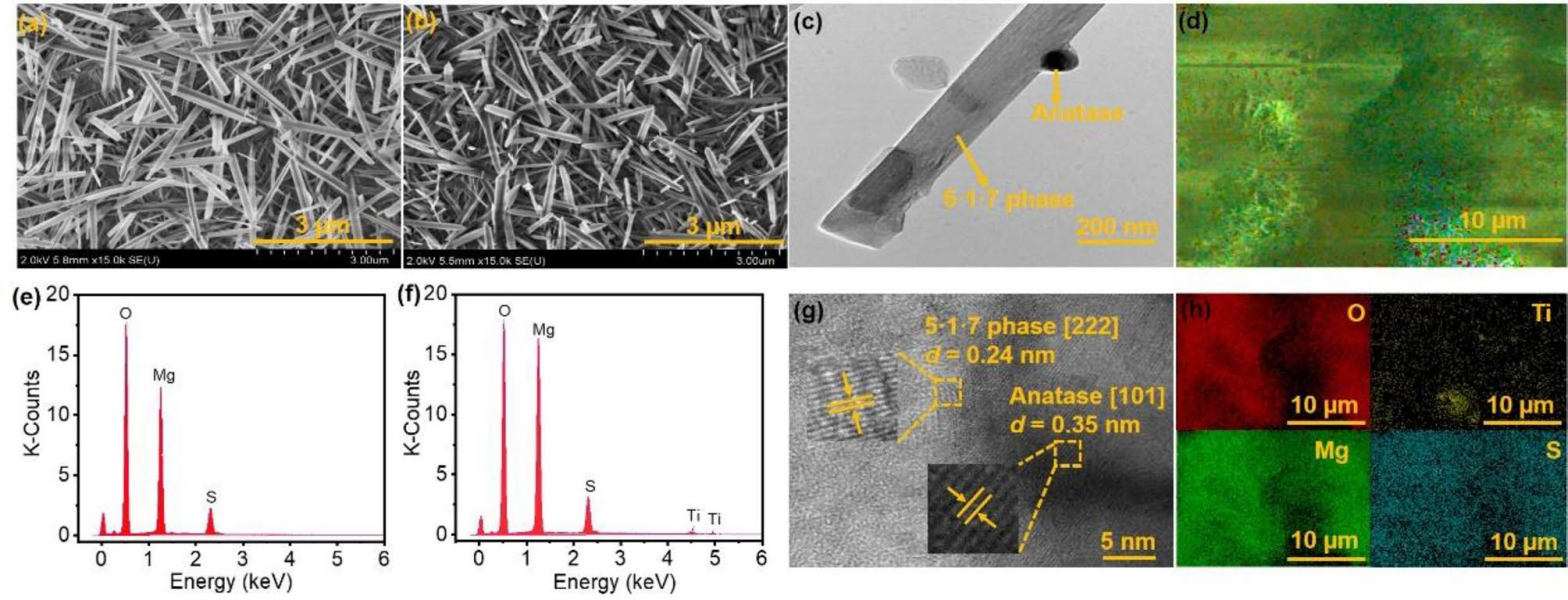
(**a**,**b**) SEM images of BMS and BMS@TiO_2_, (**c**,**g**) TEM images of BMS@TiO_2_, (**e**,**f**) EDS spectra of BMS and BMS@TiO_2_, (**d**,**h**) EDS mapping images of BMS@TiO_2_.

The morphology of BMS and BMS@TiO_2_ were characterized by SEM and TEM shown in Fig. 2. Both BMS and BMS@TiO_2_ particles are mainly composed of irregular-crisscross whiskers, and their morphology is consistent with that of the 5·1·7 phase (5Mg(OH)_2_·MgSO_4_·7H_2_O) formed in the vent of the basic magnesium sulfate cement block^43^. Additionally, TiO_2_ nanoparticle is combined with 5·1·7 phase, as evidenced by the lattice stripes of 5·1·7 phase and TiO_2_ corresponding to the (222) and (101) planes respectively shown in Fig. 2g, which also demonstrates that TiO_2_ can be firmly embedded in BMS substrate by the cement reactions between magnesium oxide and magnesium sulfate at room temperature. Figure 2e and f display the element distribution of BMS and BMS@TiO_2_, and element composition is listed in Table S2. Obviously, TiO_2_ is widely distributed in 5·1·7 phase matrix and enriched in some regions probably because of the inevitable agglomeration of TiO_2_ nanoparticles during the synthesis process of BMS@TiO_2_. The content of titanium (Ti) in BMS@TiO_2_ is 1.4% and the mass fraction of TiO_2_ in BMS@TiO_2_ is further calculated to be 2.3% approximately.

#### Physical properties and structural characteristics of BMS@TiO_2_

**Figure 3:**
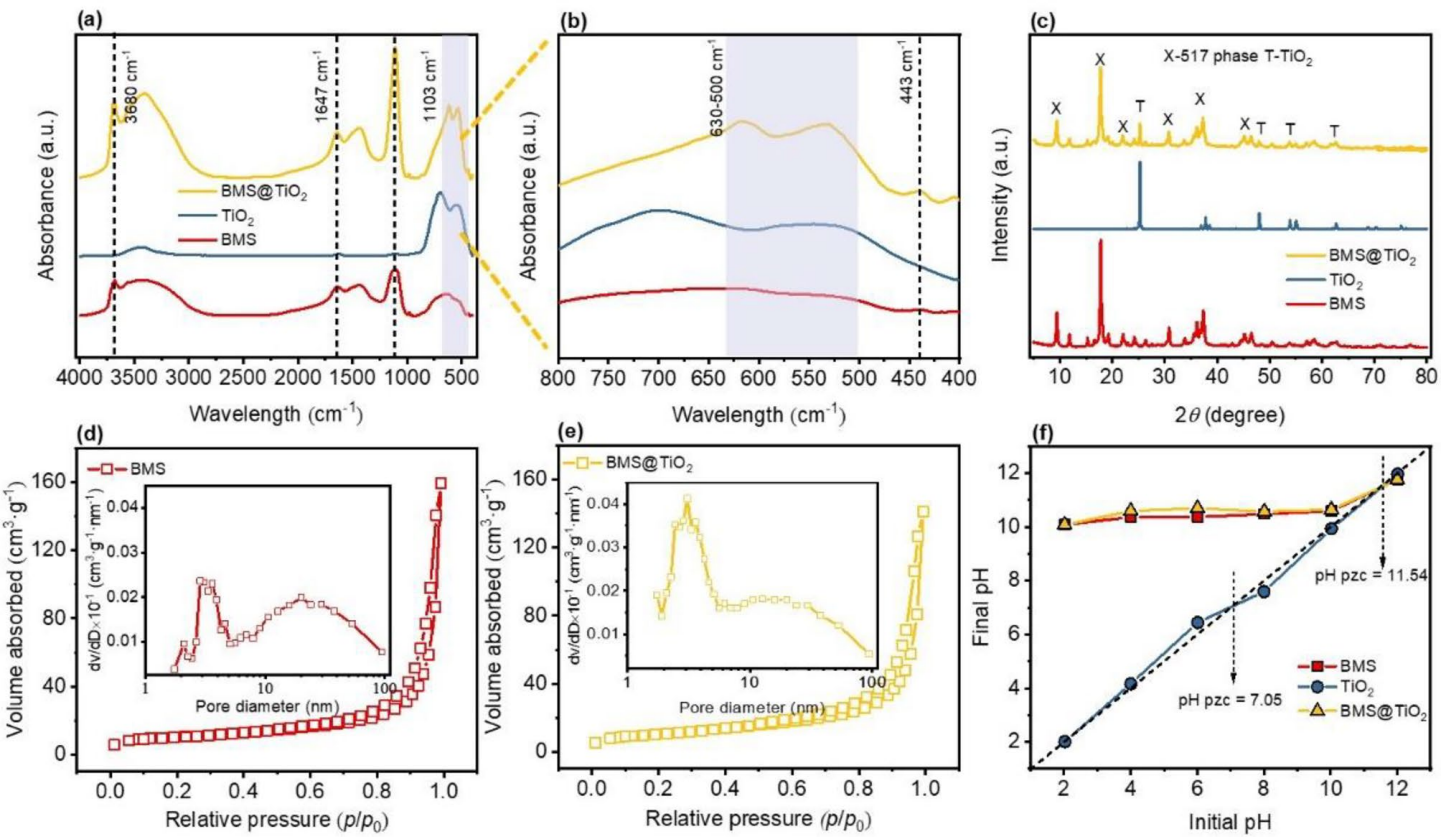
(**a**) and (**b**) FTIR spectra of BMS, TiO_2_ and BMS@TiO_2_, (**c**) XRD patterns of BMS, TiO_2_ and BMS@TiO_2_, (**d**,**e**) N_2_ adsorption–desorption isotherms and pore size distributions of BMS, and BMS@TiO_2_, (**f**) Point of zero charge (PZC) analysis of BMS, TiO_2_, and BMS@TiO_2_.

Figure 3a and b show the FTIR spectra of BMS, TiO_2_, and BMS@TiO_2_. In the spectrum of TiO_2_, the vibration band at 3438 cm^−1^ indicates the presence of residual H_2_O molecules adsorbed on TiO_2_. Meanwhile, the stretching vibration peak at 1618 cm^−1^ corresponds to the bending vibration of O-H groups^44^. The strong band at 1012 cm^−1^ indicates the vibration of the Ti-O-Ti or Ti–O bond^45^. Furthermore, the vibration peak of Ti-O-Ti at 450-750 cm^−1^ is the anatase phase of TiO_2_^46,47^. In BMS and BMS@TiO_2_, the peak at 3700 cm^−1^ occurs the stretching vibration of OH^−^, and the broad band peak at 3400 cm^−1^ is caused by the stretching vibration of crystal water (H-O), while the peak at 1636 cm^−1^ is due to the bending vibration of crystal water. The absorption band at 1450 cm^−1^, which also appears in the infrared spectrum of magnesium hydroxide^48^, probably corresponds to the asymmetric stretching vibration peak of Mg-OH. Likewise, the peak at 1103 cm^−1^ corresponds to the O_3_S-O stretching vibration peak of SO_4_^2−^. The peak at 617 cm^−1^ represents the stretching vibration peak of the S-O bond, while the subtle peak at 443 cm^−1^ corresponds to the stretching vibration peak of MgO-H^49–51^, as well as the distinctive stretching vibration peak of Ti-O appears at 530 cm^−1^ in the BMS@TiO_2_ spectrum. Furthermore, XRD patterns of BMS, TiO_2_, and BMS@TiO_2_ in Fig. 3c also indicate that the predominant component both in BMS and BMS@TiO_2_ is 5·1·7 phase, and the characteristic peaks at 9.44º, 17.80º, 30.83º, 36.15º and 37.34º belong to the distinctive features of 5·1·7 phase^52–54^. Besides, TiO_2_ in BMS@TiO_2_ exhibits distinct anatase features with characteristic peaks at 25.33º, 36.95º, 37.88º, 38.59º, 48.07º, 53.88º, 55.15º, 62.71º, as described in previous studies^45,55^.

The hysteresis characteristic and pore properties of BMS and BMS@TiO_2_ were assessed using the N_2_ adsorption-desorption method, and the results are depicted in Fig. 3d and e. It can be proved that the hysteresis loops of BMS and BMS@TiO_2_ exhibit typical IV isotherms with H3 hysteresis loops. This phenomenon possibly arises from the formation of slit-like pores among 5·1·7 phase whisker clusters^56^. The pore size distribution curves show that the major pore at 2.5-3.5 nm accompanied at approximately 20 nm appear in BMS and BMS@TiO_2_, and the specific surface area of BMS@TiO_2_ is about 37 m^2^ g^−1^ similar to that of BMS presented in Table S3. It is conceivable that the macroscopical gas hole formed during the foaming step by hydrogen peroxide and the microcosmic slit-like pores formed by 5·1·7 whisker clusters endow BMS@TiO_2_ with a large specific surface area, which is beneficial to the adsorption and photocatalysis processes. The point of zero charges (PZC) of BMS, TiO_2_, and BMS@TiO_2_ were measured by comparing the solution initial pH and final pH shown in Fig. 3f, and the pH_PZC_ values of BMS, TiO_2_, and BMS@TiO_2_ are 11.54, 7.05, and 11.54, respectively. The physical properties and structural characteristics of basic magnesium sulfate material exhibited negligible alteration following the incorporation of TiO_2_.

#### Optical properties of BMS@TiO_2_

**Figure 4:**
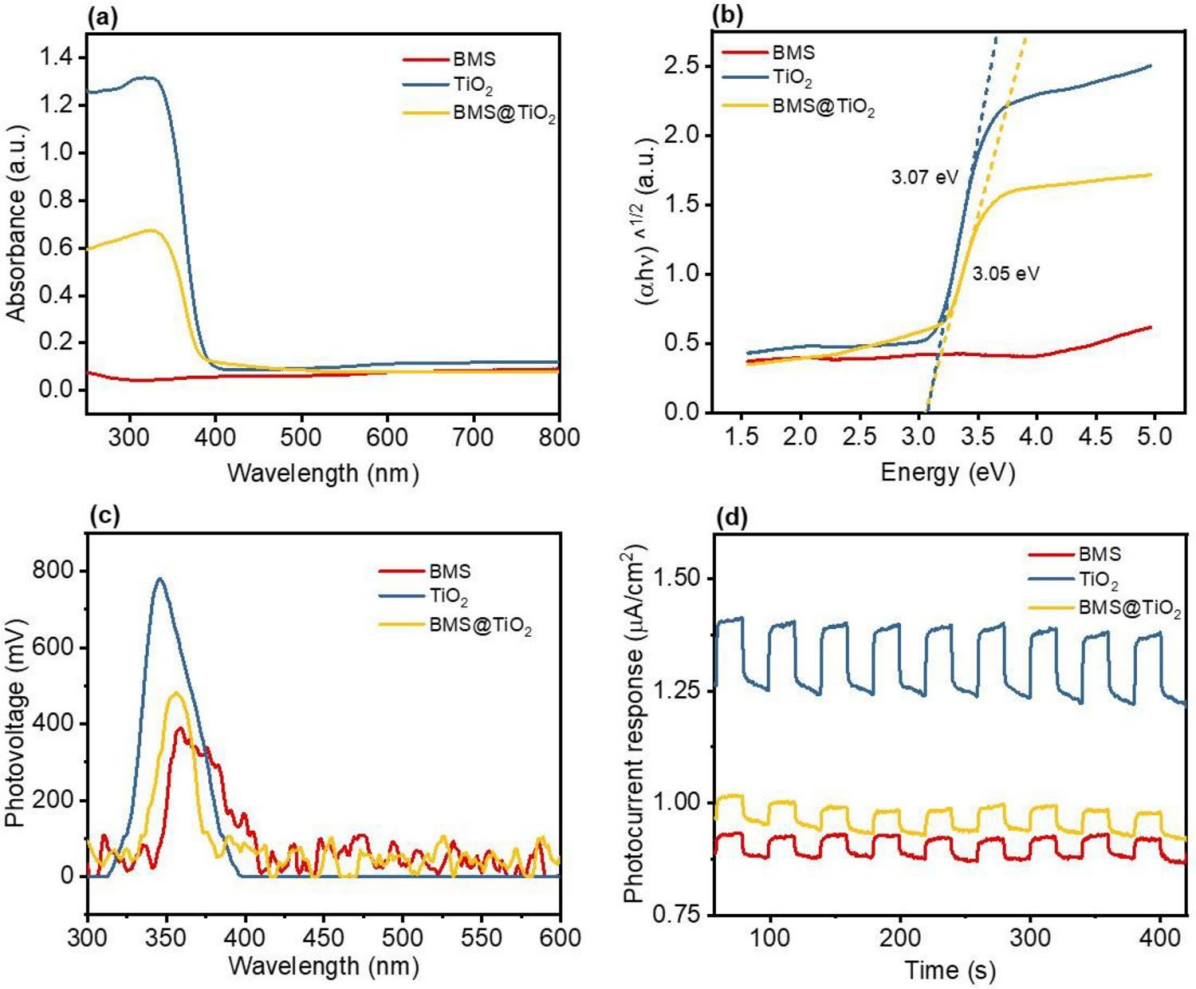
(**a**) UV-DRS spectra, (**b**) Tauc plots analysis, (**c**) Surface photovoltage spectra, and (**d**) Transient photocurrent density spectra of BMS, TiO_2_, and BMS@TiO_2_.

The optical properties of BMS, TiO_2_, and BMS@TiO_2_ were evaluated using UV diffuse reflection spectroscopy (UV-DRS) and photoelectric signal detection, and the results are presented in Fig. 4. Compared with TiO_2_, BMS@TiO_2_ also exhibits enhanced light absorption responses within 250-450 nm, and the absorbance exceeds 0.6, which is up to the half of pure TiO_2_. The band gap between the conduction and valence bands of the material is determined by the Tauc plots, as shown in formula (6)^57^.

6 $$(\alpha h\nu )^{n} = A(h\nu - E_{g})$$

where *α* is the absorbance value (a.u.), *h* is Planck's constant, *v* is the optical frequency, *E*_*g*_ is the band gap energy (eV), and *A* is the constant. The value of *n* depends on the type of semiconductor material. TiO_2_ (anatase) is an indirect transition semiconductor material, so the *n* value is 1/2.

Figure 4b illustrates that BMS@TiO_2_ has a band gap energy at about 3.05 eV, similar to TiO_2_, and can be excited by ultraviolet light. Meanwhile, the steady-state surface photovoltage and transient photocurrent density are used to study the photoelectric conversion efficiency, that is, the separation and transfer efficiency of photogenerated charge and hole. In general, the higher the photovoltage and photocurrent, the stronger the photogenerated carrier transfer ability^58^. Figure 4c and d show that all BMS, TiO_2_, and BMS@TiO_2_ exhibits a certain photoelectric conversion ability, with the order of strength being TiO_2_ > BMS@TiO_2_ > BMS. As evidenced by Fig. 4, adding a small amount of TiO_2_ (2.3%) significantly improved the photoelectric properties of basic magnesium sulfate, and thus BMS@TiO_2_ has an appropriate photocatalytic ability.

### Adsorption study

**Figure 5:**
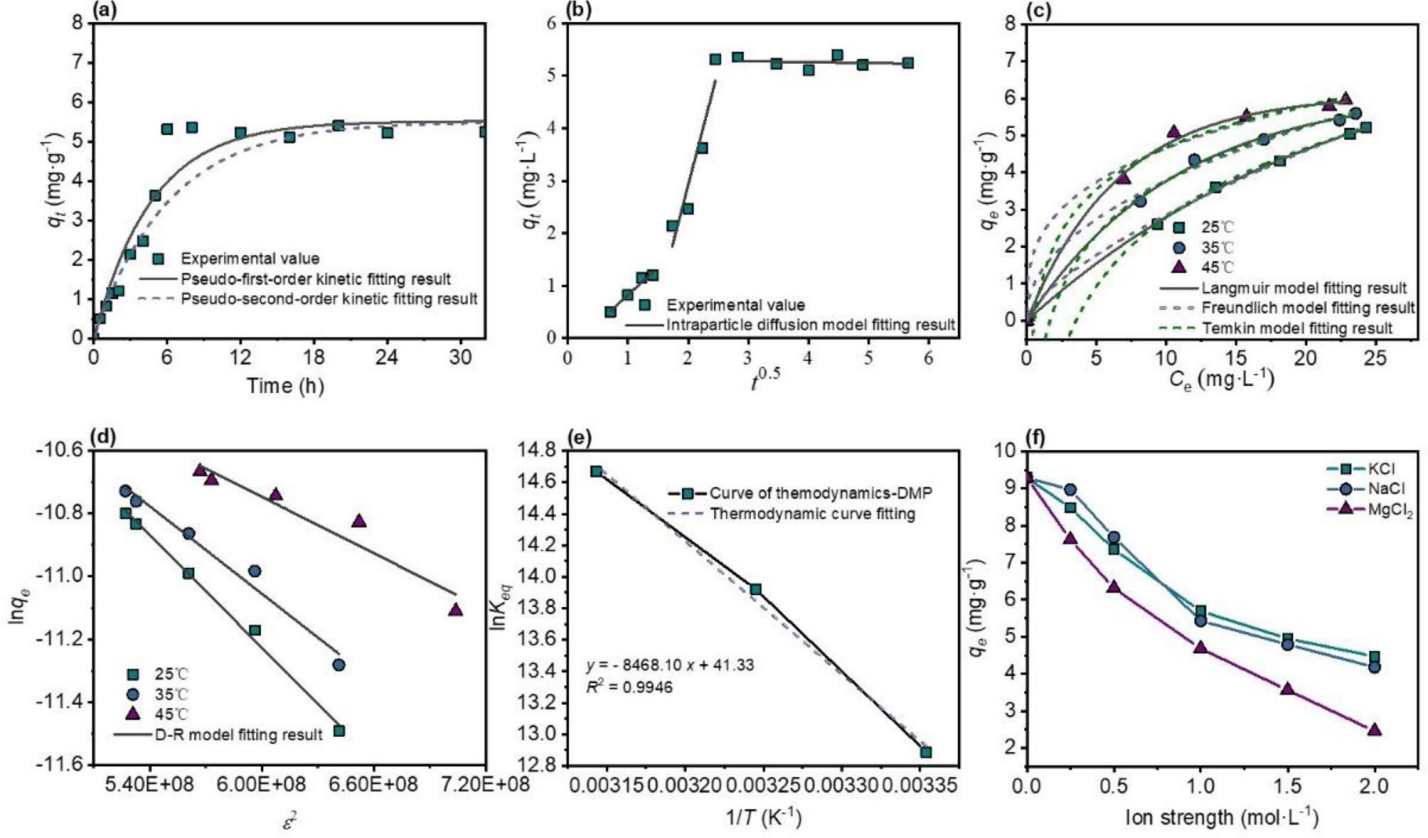
Kinetic date fitting of (**a**) pseudo-first-order, and pseudo-second-order, (**b**) intraparticle diffusion (*C*_0_ = 30 mg L^−1^, *T* = 25 °C, *I*_NaCl_ = 1 mol L^−1^, pH = 6.17), isothermal date fitting of (**c**) Langmuir, Freundlich, and Temkin, (**d**) Dubinin-Radushkevich, (**e**) Van’t Hoff equation plot (ln *K*_*eq*_ vs. 1/*T*) to determine the thermodynamics parameter, (**f**) effect of co-existing salts.

Figure 5a and b show the kinetics of DMP adsorption by BMS@TiO_2_. The adsorption equilibrium is reached after 6 h, and the adsorption capacity is maintained at approximately 5.32 mg g^−1^ in 1 mol L^−1^ NaCl solution with an initial DMP concentration of 30 mg L^−1^. The model fitting parameters are shown in Table S4. The *R*^2^ value of the pseudo-second-order fitting is higher than that of other models, and the theoretical maximum adsorption value of 5.51 mg g^−1^ is close to the experimental value. Therefore, the adsorption kinetics of BMS@TiO_2_ for DMP can be accurately described by a pseudo-second-order model. Particle diffusion is also used to explain the rate-limiting step of the process. The multi-step control process of adsorption is represented by the relationship between *q*_*e*_ and t^0.5^. If a linear plot passing through the origin is obtained, it is presumed that adsorption occurs solely through intraparticle diffusion. If a linear graph is obtained that does not pass through the origin, the process is controlled by two or more steps^59,60^. The graphs obtained in this study show linear relationships that do not intersect at the origin. Thus, these results show that although adsorption occurs through intraparticle diffusion, this is not the only rate-controlling step of the process.

The adsorption effect of BMS@TiO_2_ on DMP at three different temperatures of 298.15 *K*, 308.15 *K*, and 318.15 *K* was investigated. Figure 5c and d show the fitting of Langmuir, Freundlich, Temkin, and Dubinin-Radushkevich isotherm models to the data, with specific fitting parameters presented in Tables S5 and S6. The model that best describes the equilibrium data was selected based on the highest *R*^2^ value. According to the parameters in Tables S5 and S6, the Langmuir and Temkin models exhibited similar and the best fitting *R*^2^ values, followed by the Freundlich and Dubinin-Radushkevich models. The good fit of Langmuir and Temkin isotherm models indicates a monolayer chemical adsorption process, while the Dubinin-Radushkevich isotherm model is used to explain the influence of the adsorbent's porous structure. In this model, the adsorption process is related to the filling of micropore volume rather than layer-by-layer adsorption on the pore walls^61,62^. The Dubinin-Radushkevich model parameter *B*_*DR*_ can be used to estimate the average free energy ($E{ = 1/}\sqrt {{2}B_{DR} }$) and distinguish between different types of adsorption processes. When the value of *E* is less than 8 kJ mol^−1^, the adsorption process is physical adsorption; when *E* is between 8 kJ mol^−1^ and 16 kJ mol^−1^, the process is chemical adsorption^63^. The results in Table S6 show that the value of *E* ranges between 8 and 16 kJ mol^−1^, indicating that BMS@TiO_2_ on DMP involves a single molecular layer chemical adsorption process. Furthermore, the Langmuir equilibrium constants in milligrams are used in the Van't Hoff equation, and the values of Δ*G*, Δ*H*^o^, and Δ*S*^o^ are obtained from Fig. 5e and summarized in Table S7. The negative Δ*G* values for different temperatures and the positive Δ*H*^o^ value indicates that the adsorption process is endothermic and spontaneous.

Furthermore, the influence of different co-existing salts and ionic strengths on the adsorption capacity is illustrated in Fig. 5f. The general trend is that the adsorption capacity decreases with the increase of ionic strength. With the same ionic strength, the adsorption capacity of potassium chloride and sodium chloride changes to a similar extent. According to XRD patterns in Fig. S2, the phase composition of BMS@TiO_2_ remains unchanged in both pure water and brine solution, exhibiting certain resistance to water and salt^64,65^. There seems to because co-existing salts may affect the adsorption process, which in turn leads to a decrease in adsorption capacity.

Based on the porous structure characteristics of BMS@TiO_2_, it is possible that DMP can be adsorbed through pore interception. In this process, cations compete with water molecules for DMP, thereby reducing the affinity between DMP and water molecules. This makes DMP more prone to aggregation, ultimately lowering the critical micelle concentration (CMC) of DMP. Therefore, the aggregated DMP is less likely to be absorbed onto BMS@TiO_2_ through pore interception, reducing the probability of interacting with BMS@TiO_2_ through mutual adsorption. In addition, compared to Na^+^ and K^+^, Mg^2+^ has a stronger hydration ability, leading to more pronounced salting-out effects. Furthermore, the decrease in the critical micelle concentration (CMC) value of DMP is more significant after the addition of Mg^2+^^66,67^.

Figure S3 shows SEM images, XRD patterns, FTIR spectra, and XPS spectra for BMS@TiO_2_ before and after adsorption. The results indicate that in BMS@TiO_2_ after adsorption, the 5·1·7 phase structure is still maintained, and regular whisker shape in SEM images are no longer present, which may be caused by the absorption of DMP onto 5·1·7 phase. Meanwhile, the corresponding FTIR spectra characteristic peaks of 5·1·7 phase do not significantly change before and after absorption, and the peaks at 2927 cm^−1^ and 2851 cm^−1^ appeared in the sample after adsorption are respectively assigned to the asymmetric (*ν*_a_) and symmetric (*ν*_s_) stretching modes of -CH_2_- groups in the organic adsorbate^68^. After adsorption, the broad peak intensity around 3400 cm^−1^ increases, and the peak around 1650 cm^−1^ shifts, possibly due to the formation of hydrogen bonds between BMS@TiO_2_ and DMP^69^. Analysis of the O1s spectrum in Fig. S3e shows that the peak O_α_ at around 531.1 eV is attributed to lattice oxygen, and the peak O_β_ at around 531.5 eV is attributed to hydroxyl oxygen. After adsorption, the proportion of hydroxyl oxygen increases from 42.65 to 47.2%, and the binding energy of hydroxyl oxygen decreases from 531.5 eV to 531.26 eV, indicating an increase in electron density around hydroxyl oxygen. These results suggest that BMS@TiO_2_ may adsorb DMP through hydrogen bonding^70,71^.

### Photocatalytic degradation behavior and kinetic evaluation of DMP

**Figure 6:**
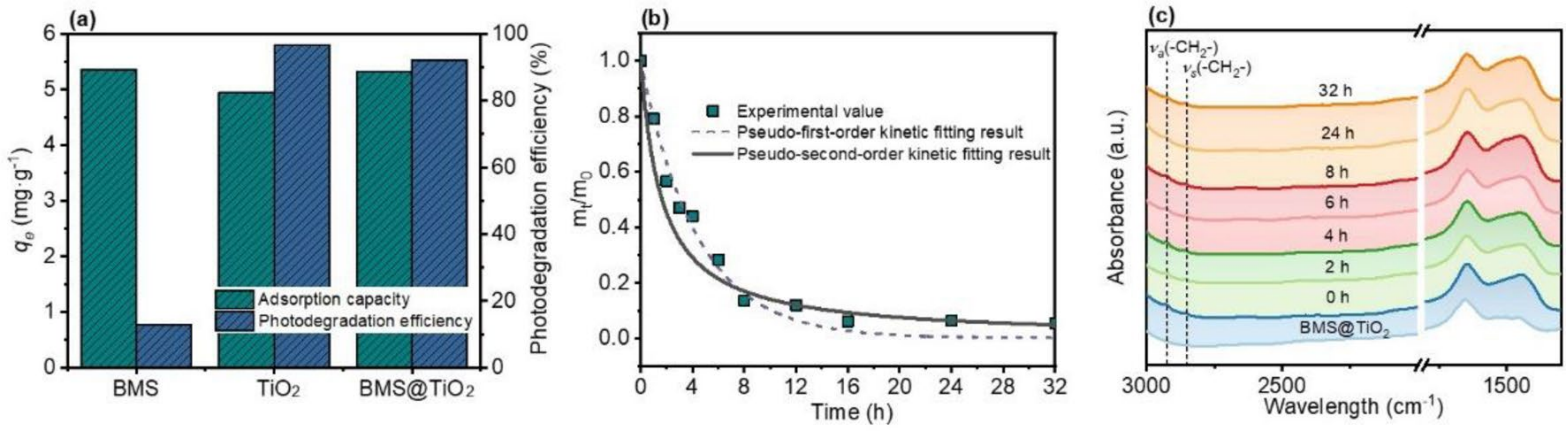
(**a**) Adsorption capacity and photodegradation efficiency of DMP on BMS, TiO_2_, and BMS@TiO_2_, (**b**) photodegradation kinetics, and (**c**) FTIR spectra of BMS@TiO_2_ before and after photodegradation at different time.

Figure 6a demonstrates the adsorption capacity and photodegradation efficiency of DMP on BMS, TiO_2_, and BMS@TiO_2_. Compared with TiO_2_ nanoparticles, 20-60 mesh BMS@TiO_2_ particles with 2.3% TiO_2_ exhibits an equally excellent adsorption and photocatalytic performance. Additionally, the photodegradation efficiency of DMP adsorbed on BMS particles reaches about 12%, which reveals that BMS also has weak photocatalytic activity, and this may be because BMS has weak light absorbance and some degree of photoelectric conversion efficiency shown in Fig. 4. Pseudo-first-order and pseudo-second-order kinetic models are employed to fit the photodegradation kinetics data of DMP on BMS@TiO_2_ in Fig. 6b, and the corresponding fitting parameters are listed in Table S8. The results indicate that the photodegradation equilibrium of DMP is reached after 16 h and the photodegradation efficiency of DMP is approximately 92%. And photocatalytic kinetics of DMP on BMS@TiO_2_ can be accurately represented by a pseudo-first-order kinetic model.

In addition, as depicted in Fig. 6c, FTIR spectra of BMS@TiO_2_ before and after photodegradation at different time are obtained to verify the photodegradation kinetics behavior of DMP. The intensity of the peaks at 2927 cm^−1^ and 2851 cm^−1^ assigned to the asymmetric (*ν*_a_) and symmetric (*ν*_s_) stretching of -CH_2_- groups of the organic adsorbate gradually decreases with the extension of photodegradation time, adequately manifesting the photodegradation and removal of DMP adsorbed on BMS@TiO_2_. The total organic carbon (TOC) removal efficiency and GC-MS analysis of adsorbates on BMS@TiO_2_ before and after photodegradation at different times shown in Fig. S4 are employed to further understand the photodegradation behavior of DMP. As the photodegradation time extends, TOC removal efficiency is up to 52% within 16 h, distinctly lower than the photodegradation efficiency of DMP. According to the GC-MS analysis results of adsorbates on BMS@TiO_2_ before and after photodegradation at 12 and 16 h, there are at least three intermediates in industrial DMP, herein identified as I1, I2, and I3, respectively^72^. Moreover, DMP on BMS@TiO_2_ thoroughly vanished by ultraviolet radiation at 16 h, while the contents of these impurities slowly diminish. It is reasonable to conclude that DMP adsorbed on BMS@TiO_2_ can be degraded and a small portion of impurities can also be adsorbed and photodegraded to varying degrees may because of their different degradability, limiting the apparent TOC removal efficiency.

### Stability and reusability of BMS@TiO_2_

**Figure 7:**
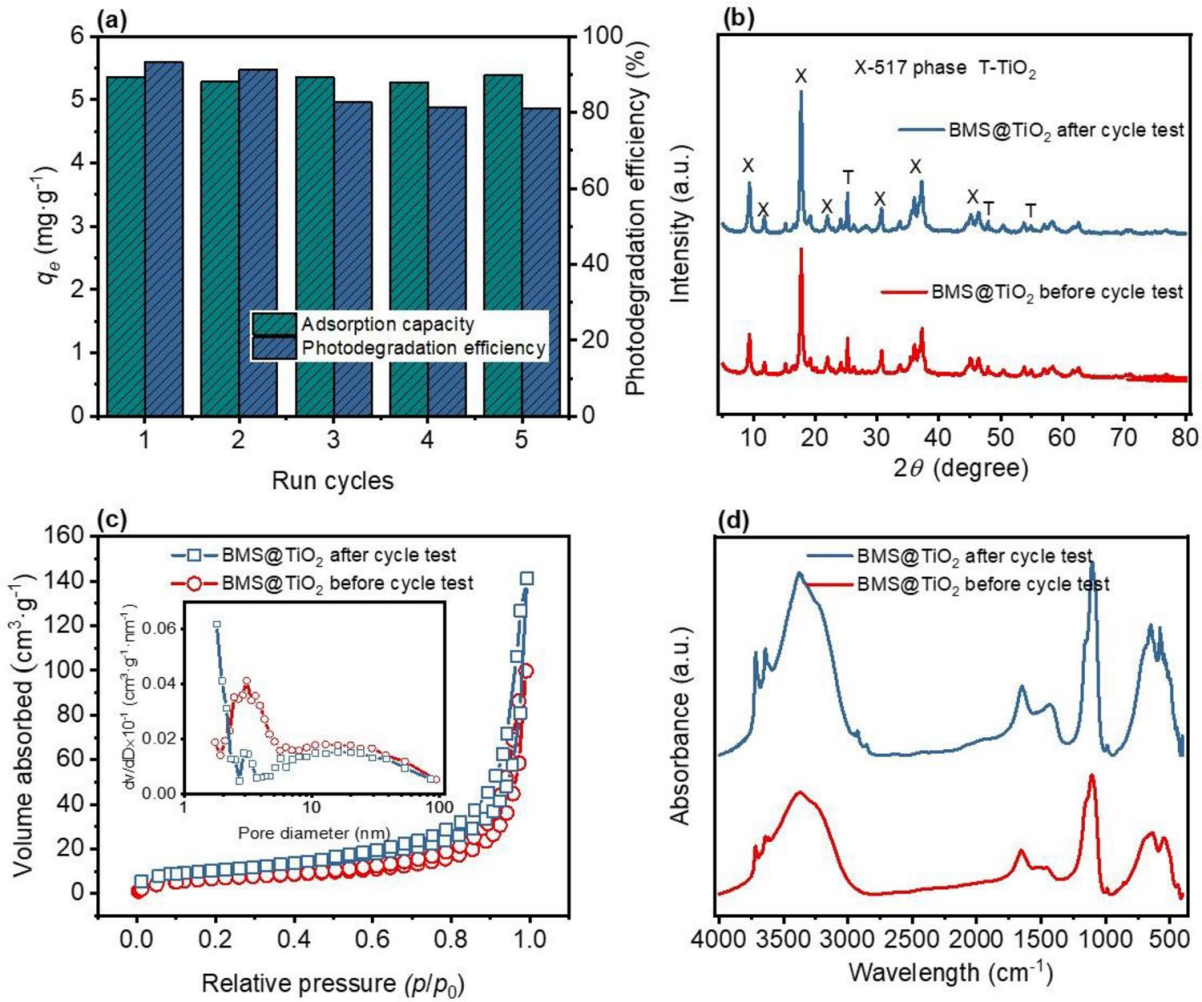
(**a**) The cycle test of adsorption and photocatalytic performance of BMS@TiO_2_ for removing DMP, (**b**) XRD patterns, (**c**) N_2_ adsorption and desorption hysteresis loops, and (**d**) FTIR spectra of BMS@TiO_2_ before and after cycle tests.

The consecutive adsorption and photodegradation tests for DMP on BMS@TiO_2_ are conducted, and BMS@TiO_2_ samples before and after cycle tests are analyzed by XRD, FTIR and N_2_ adsorption/desorption method. The results obtained in Fig. 7 reveal that BMS@TiO_2_ maintains a consistent adsorption performance with an average adsorption capacity of 5.33 mg g^−1^ after five cycle tests, while the photocatalytic degradation efficiency gradually decreases from 92% at the first cycle to 81% at the fifth cycle. XRD patterns and pore characteristics of BMS@TiO_2_ before and after cycle tests evidence that all of 5·1·7 phase, TiO_2_ and the slit-pores at 2.5–3.5 nm accompanied at approximately 20 nm always exists, demonstrating that electrostatic interaction, pore interception and later photocatalysis of BMS@TiO_2_ interacted with DMP steadily accomplished during cycle tests. Figure 7d shows the FTIR spectra of BMS@TiO_2_ before and after cycle tests. The corresponding characteristic peaks of BMS@TiO_2_ remain unchanged, and peaks at 2927 cm^−1^ and 2851 cm^−1^ attributed to the asymmetric (*ν*_a_) and symmetric (*ν*_s_) stretching patterns of -CH_2_- groups appear after cycle tests. This is because, the residual impurities adsorbed on BMS@TiO_2_ compete to consume active radicals in the next photodegradation, resulting in a decrease in the next apparent photocatalytic degradation efficiency. The degradation efficiency remained at approximately 81% in the third to fifth cycles shown in Fig. 7a, which may be due to the residual quantity of impurities on BMS@TiO_2_ reaching adsorption-degradation equilibrium at the cycle experimental conditions.

### Photodegradation mechanism of DMP by BMS@TiO_2_

**Figure 8:**
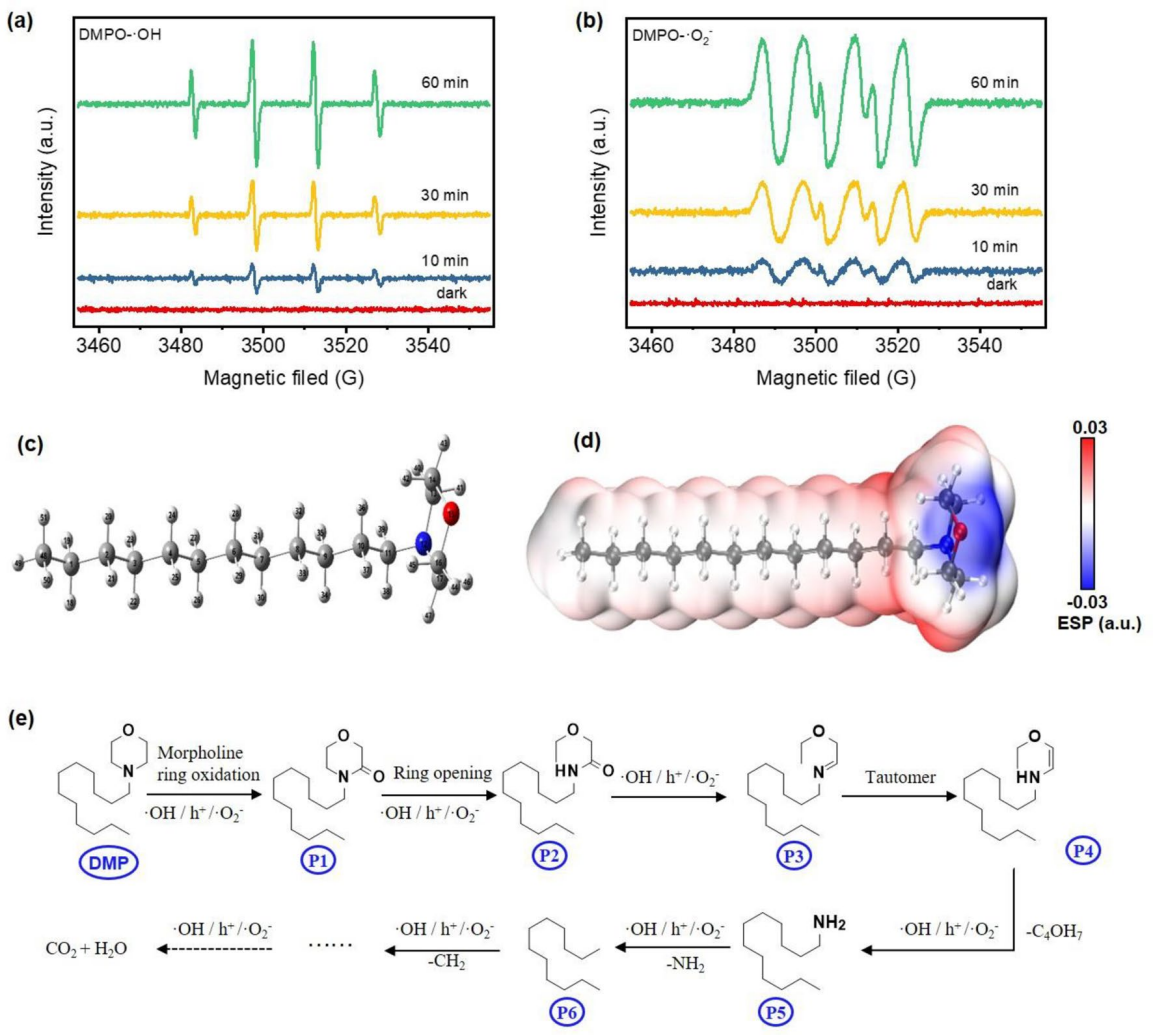
(**a**) EPR spectrum of DMPO-⋅OH, and (**b**) EPR spectrum of DMPO-⋅O_2_^−^ in BMS@TiO_2_ photocatalytic system, (**c**) Optimized structure of DMP at B3LYP-D3BJ/def2-SVPlevel, (**d**) molecular electrostatic potential of DMP, (**e**) Proposed photodegradation pathways of DMP by BMS@TiO_2_ under UV irradiation.

EPR spectra are conducted using 5,5-dimethyl-1-pyrroline N-oxide (DMPO) as the spin trapping agent in order to track the generation process of active species for degrading DMP adsorbed on BMS@TiO_2_. The remarkable characteristic peaks in Fig. 8a and b indicate respectively the formation of ·OH and ·O_2_^−^ in BMS@TiO_2_ photocatalytic system, which illustrates that the photodegradation removal of DMP adsorbed on BMS@TiO_2_ is mainly accomplished by ·OH and ·O_2_^−^. And the signal intensity of all peaks gradually increases with the extension of exposure time, indicating the continuous generation of active species. The charge distribution and reactive sites of DMP are revealed by the electrostatic potential. This potential predicts the nucleophilic and electrophilic regions of the molecule, and negative and positive electrostatic potential regions favoring the occurrence of electrophilic attacks and nucleophilic attacks, respectively^73,74^. As shown in Fig. 8d, the electrostatic potential of DMP is visualized as red and blue surfaces surrounding the molecule. The blue color represents negative electrostatic potential values and the red color represents positive electrostatic potential values. The negative electrostatic potential region (blue colored) can be observed for the nitrogen and oxygen atoms located on the morpholine ring, and the positive electrostatic potential region (red colored) is located on the morpholine ring and the hydrogen atoms on C10 and C11 (Fig. 8c). Therefore, the nitrogen and oxygen atoms on the morpholine ring are susceptible to react with photogenerated holes and radicals, while the hydrogen atoms on the ring are susceptible to nucleophilic reaction.

Based on the electrostatic potential of DMP and the intermediates, the possible reaction mechanism and degradation pathways of DMP by BMS@TiO_2_ are proposed in Fig. 8e. It is speculated that the hydrogen atoms on C17 of DMP are oxidized by the photogenerated radicals through nucleophilic reaction. As a result, the -CH_2_- group on the ring was oxidized and transformed into the C = O group^75^. Next, the generated P2 is oxidized by radicals h^+^, ⋅OH, and ⋅O_2_^−^ to produce P3, which underwent rapid transformation to P4 through tautomerization. Groups -C_4_OH_7_ in P4 are removed by radicals to form a long-chain alkane primary amine (P5)^76^. The primary amine (P5) is then progressively deaminated and demethylated to form P6 by radicals. Ultimately, these intermediate products may be broken down into small molecules and completely mineralize into CO_2_ and H_2_O. The lack of inclusion of intermediates in the GC–MS at 12 and 16 h is probably owing to their poor stability and susceptibility to degradation in the experimental conditions.

**Figure 9:**
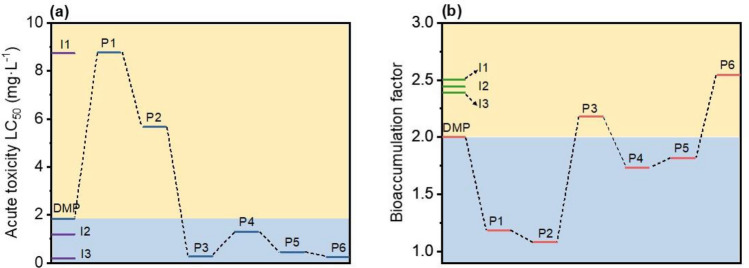
(**a**) Acute toxicity of the fathead minnow LC_50_-96 h, and (**b**) bioaccumulation factor of DMP and degradation intermediates predicted by T.E.S.T. based on the consensus method.

**Figure 10:**
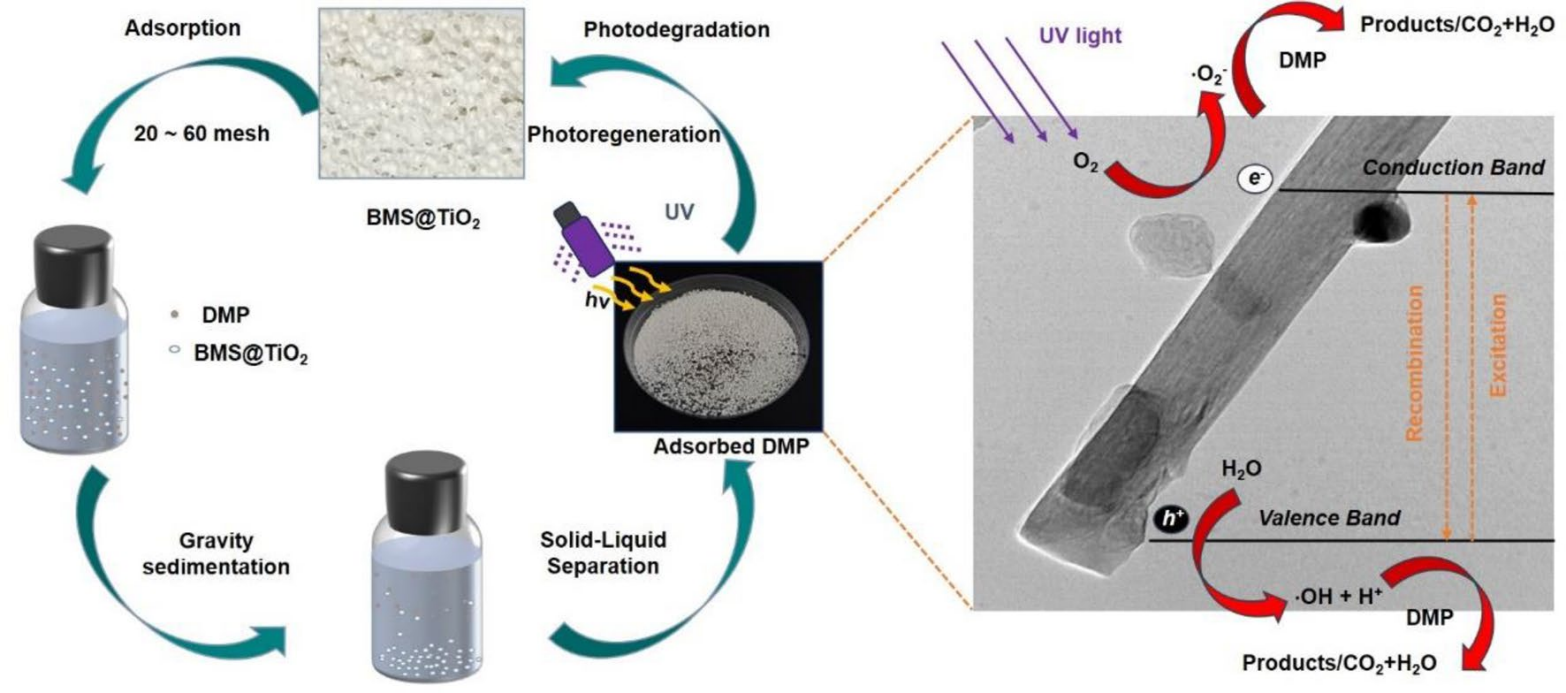
Adsorption and photodegradation process of DMP by BMS@TiO_2_.

As shown in Fig. 9, acute toxicity (as measured by the fathead minnow 50% lethal dose (LC_50_-96 h) and bioaccumulation factor are employed to evaluate the toxicities of DMP and the speculative photodegradation intermediates through the Toxicity Estimation Software Tool (T.E.S.T.) using the consensus method based on Quantitative Structure Activity Relationship (QSAR) prediction. Compared to DMP, the acute toxicities of intermediates tend to decrease when generating P1 and P2, then increase up until ensuing P3-P6, signifying that most intermediates are unfavored for reducing the toxicity and potential danger to the aqueous environment. The bioaccumulation factor exhibits similar pattern as acute toxicity, implying that only sufficient photocatalytic degradation and entire mineralization of DMP to CO_2_ and H_2_O could alleviate the bioconcentration effect of DMP on the environment. Simultaneously, the impurities in industrial DMP, except I1, are more toxic and tendentious than DMP to bioconcentration effect. It is conceivable that the treatment procedures for adsorption of DMP and coexisting harmful impurities from brine and then photocatalytic degradation by BMS@TiO_2_ in air could be an efficient and environmentally friendly approach to removing micropollutants, avoiding harmful intermediates enter the brine and cause secondary pollution. Figure 10 summarizes the process of adsorption and photodegradation of DMP using BMS@TiO_2_.

## Conclusion

In summary, the adsorption and photocatalytic degradation performances of BMS@TiO_2_ composite for removing DMP from brine are explored thoroughly in this work. TiO_2_ is firmly embedded in porous BMS substrate at room temperature with a loading content of 2.3%, as illustrated by the TEM images, the lattice stripe analysis, and the EDS results, respectively. In BMS@TiO_2_ composite, the macroscopical gas hole formed during the foaming step by hydrogen peroxide and the microcosmic slit-like pores formed by 5·1·7 whisker clusters bring a specific surface area of about 37 m^2^ g^−1^, and it is advantageous to adsorption and photocatalysis. The adsorption of DMP on the surface of BMS@TiO_2_ composite is realized through hydrogen bonding and pore interception, which can be revealed according to XPS, FTIR, coexisting salt effect, and SEM images. The isotherm and kinetics analysis indicates that the adsorption of DMP on BMS@TiO_2_ involves a single molecular layer chemical adsorption process, which is essentially spontaneous and endothermic. Depending on the DRS, degradation efficiency, FTIR, and GC-MS analysis results, it is concluded that BMS@TiO_2_ composite presents a similar photocatalytic degradation capability to pure TiO_2_ powder with a band gap of 3.05 eV, and the degradation efficiency of DMP reaches 92% by direct UV irradiation for 16 h. Five consecutive cycles of adsorption and photocatalytic degradation experiments confirm that BMS@TiO_2_ composite exhibits excellent absorption and photodegradation performance and reusability. EPR spectra results verify the unremitting generation of radicals ⋅OH and ⋅O_2_^−^ on BMS@TiO_2_ interface by direct UV irradiation. Furthermore, the active reaction sites of DMP molecule are predicted by the electrostatic potential distribution calculation, and the photocatalytic degradation pathway is supposed to describe the photodegradation behavior of DMP. Therefore, our work proposes a mild approach for produce easy-recyclable and effective adsorbent and photocatalyst and safe removal treatment of DMP from brine.

### Supplementary Information

Supplementary Information.

## Data Availability

The datasets used and/or analysed during the current study available from the corresponding author on reasonable request.
