# Supplementary material for: Basic magnesium sulfate@TiO2 composite for efficient adsorption and photocatalytic degradation of 4-dodecylmorpholine in brine
Source: Sci Rep. 2024 Apr 23;14:9315. doi: 10.1038/s41598-024-59921-8 (PMC11039658; doi:10.1038/s41598-024-59921-8)
Supplement: Supplementary file 1 — Supplementary Information. [file 41598_2024_59921_MOESM1_ESM.docx]

**Basic magnesium sulfate@TiO_2_ composite for efficient** **adsorption and photocatalytic degradation of 4-dodecylmorpholine in brine**

Zhongmei Song ^a,d^, Huifang Zhang ^a,^[[1]](#footnote-1)^*^, Liang Ma^a,d^, Miao Lu^a,d^, Chengyou Wu^b,^[[2]](#footnote-2)^*^, Qingqing Liu ^c^, Xuefeng Yu ^c^, Haining Liu ^a,^[[3]](#footnote-3)^*^, Xiushen Ye ^a^, Zhen Ma ^c^, Zhijian Wu^a^

*^a^* *Key Laboratory of Green and High-end Utilization of Salt Lake Resources, Qinghai Institute of Salt Lakes, Chinese Academy of Sciences, Xining 810008, China*

*^b^ Qinghai University, Xining 810016, China*

*^c^ Qinghai Salt Lake Industry Co., Ltd., Golmud 816000, China*

*^d^ University of Chinese Academy of Sciences, Beijing 100049, China*

**Contents:**

**Text sections:**

**S1. Adsorption isotherms**

**S2. Adsorption kinetics**

**S3. Adsorption thermodynamics**

**S4. Density functional theory (DFT) calculation methods**

**Tables: 8**

**Figures: 4**

**S1. Adsorption isotherms**

The adsorption isotherm of the prepared BMS@TiO_2_ was analyzed using the Langmuir, Freundlich, Temkin, and Dubinin-Radushkevich models. The mathematical expressions are shown in Eq. (S1) ~ (S4), respectively [1-3].

 (S1)

 (S2)

$q_{e}=B\ln(K_{T}C_{e})$ (S3)

$\ln q_{e}=\ln q_{max}-B_{DR}\varepsilon^{2}$ (S4)

Where *q_e_*, *q_max_*, and *C_e_* represent the equilibrium adsorption (mg·g^-1^), the maximum adsorption (mg·g^-1^), and the equilibrium concentration of DMP (mg·L^-1^), respectively. *K_L_*, *K_F_*, *n*, *K_T_*, *B*, and *B_DR_* represent Langmuir, Freundlich, Temkin, and Dubinin-Radushkevich ’s constants, respectively, and *ε* (Polanyi potential) = *RT* ln (1+1/*C_e_*).

**S2. Adsorption kinetics**

As the prediction of the adsorption speed is essential in the surface adsorption process, several kinetics models such as the pseudo-first-order, pseudo-second-order and intraparticle diffusion are applied to evaluate the kinetics behavior of BMS@TiO_2_. Eq. (S5) ~ (S7) show the mathematical expression of the pseudo-first-order, pseud-second-order and intraparticle diffusion [4, 5].

 (S5)

 (S6)

$q_{t}=k_{id}\sqrt{t}+C$ (S7)

Where *q_t_* and *q_e_* represent the DMP adsorption capacity at a certain time (mg·g^-1^) and its equilibrium adsorption capacity (mg·g^-1^), *K*_1_ (h^-1^), *K*_2_ (g·mg^-1^·h^-1^) and *K_id_* (mg g^-1^ min^0.5^) are the kinetics constants of pseudo-first order, pseudo-second order and intraparticle diffusion, respectively. *C* (mg g^-1^) is a constant value depicting the boundary layer effects, and *t* is the adsorption time (h).

**S3. Adsorption thermodynamics**

The thermodynamics parameters, such as the thermodynamic equilibrium constant (*K_eq_*), Gibbs free energy (Δ*G*), standard enthalpy of adsorption (Δ*H*°), and standard entropy of adsorption (Δ*S*°) for the removal of DMP using BMS@TiO_2_ are determined by applying the Van’t Hoff equations as expressed by Eq. (S8) ~ (S10) [6, 7].

 (S8)

 (S9)

$\Delta G=\frac{\Delta S^{\circ}}{R}-\frac{\Delta H^{\circ}}{RT}$ (S10)

Here *K_L_*, and *K_eq_* represent the Langmuir constant and the standard thermodynamic equilibrium constant of adsorption, respectively. The *K_L_* constant is simply recalculated as dimensionless by multiplying it by 55.5 (the number of moles of water per liter of solution). *M_adsorbate_* represents the molar mass of DMP. Δ*G* (kJ mol^-1^), Δ*H*° (kJ mol^-1^), and Δ*S*° (J mol^-1^·K^-1^) represent the standard Gibbs free energy, enthalpy, and entropy of adsorption. *R* (8.314 J·mol^-1^·K^-1^) is the universal gas constant, and *T* represent the absolute temperature (K).

**S4. Density functional theory (DFT) calculation methods**

The theoretical calculations were performed via the Gaussian 16 suite of programs [8]. The structure of the studied molecules was fully optimized at the B3LYP-D3BJ/def2-SVP level of theory. The solvent effect was included in the calculations using the solvation model based on the density (SMD) model. The vibrational frequencies of the optimized structures were carried out at the same level. The structures were characterized as a local energy minimum on the potential energy surface by verifying that all the vibrational frequencies were real. The Visual Molecular Dynamics (VMD) program [9] was used to plot the color-filled iso-surface graphs to visualize the molecular orbitals and the molecular electrostatic potential (MESP).

**Tables**

**Table S1.** Key physicochemical properties of dodecylmorpholine [10].

| Molecular  formula | C_16_H_33_NO | 2-D structure |  |
| --- | --- | --- | --- |
| Molecular weight  (g·mol^-1^) | 255.44 | 3-D structure | 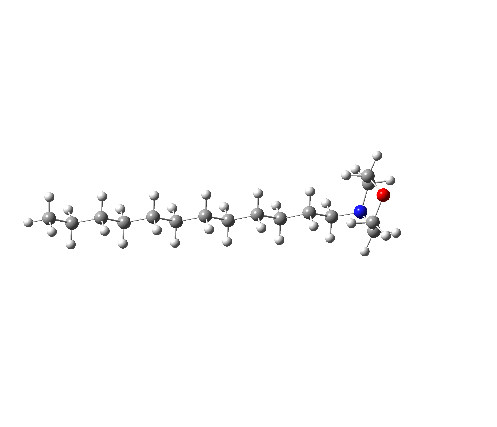 |
| Density  (25℃)  (g·cm^-3^) | 0.859 | Octanol-water partition coefficient  log *K*_ow_ | 5.05 |
| Water solubility  (25℃)  (mg·L^-1^) | insoluble | Boiling point (℃) | 321 |

**Table S2.** Elemental composition in BMS and BMS@TiO_2_.

| Elementals | Elemental measurement | | | |
| --- | --- | --- | --- | --- |
|  | BMS | | BMS@TiO_2_ | |
|  | Mass  fraction/% | Atom  fraction | Mass  fraction/% | Atom  fraction |
| Oxygen (O) | 59.3 | 0.2 | 55.3 | 0.2 |
| Magnesium (Mg) | 27.9 | 0.2 | 31.8 | 0.2 |
| Sulfur (S) | 6.9 | 0.1 | 8.3 | 0.1 |
| Carbon (C) | 5.9 | 0.3 | 3.2 | 0.3 |
| Titanium (Ti) | 0.0 | 0.0 | 1.4 | 0.1 |

**Table S3.** Pore features of BMS and BMS@TiO_2_.

| Sample | BET surface area  (m^2^·g^-1^) | Pore volume  (cm^3^·g^-1^) | Average pore  diameter (nm) |
| --- | --- | --- | --- |
| BMS | 35.30 | 0.084 | 9.53 |
| BMS@TiO_2_ | 37.24 | 0.083 | 8.95 |

**Table S4.** Kinetic model parameters of adsorption for DMP onto BMS@TiO_2_.

| Model | Parameter | Values |
| --- | --- | --- |
| Pseudo-first-order | *K_1_* (h^-1^) | 0.2128 |
|  | *q_e_* (mg·g^-1^) | 5.5131 |
|  | *R*^2^ | 0.9349 |
| Pseudo-second-order | *K_2_* (g·mg^-1^·h^-1^) | 0.1635 |
|  | *q_e_* (mg·g^-1^) | 5.4905 |
|  | *R*^2^ | 0.9907 |
| Intraparticle diffusion | *C_i,1_* (mg·g^-1^) | -0.2260 |
|  | *K_i,1_* (mg·g^-1^·h^-0.5^) | 1.0524 |
|  | *R*_1_^2^ | 0.9673 |
|  | *C_i,2_* (mg·g^-1^) | -5.8657 |
|  | *K_i,2_* (mg·g^-1^·h^-0.5^) | 4.3965 |
|  | *R*_2_^2^ | 0.8946 |
|  | *C_i,3_* (mg·g^-1^) | 5.3302 |
|  | *K_i,3_* (mg·g^-1^·h^-0.5^) | -0.0176 |
|  | *R*_3_^2^ | 0.0276 |

**Table S5.** Langmuir and Freundlich isotherm model parameters of adsorption for DMP on BMS@TiO_2_.

| Model | Temperature | Parameter | Values |
| --- | --- | --- | --- |
| Langmuir | 25℃ | *q_max_* (mg·g^-1^) | 12.8927 |
|  |  | *K_L_* (L·mg^-1^) | 0.0279 |
|  |  | *R*^2^ | 0.9995 |
|  | 35℃ | *q_max_* (mg·g^-1^) | 8.5984 |
|  |  | *K_L_* (L·mg^-1^) | 0.0784 |
|  |  | *R*^2^ | 0.9977 |
|  | 45℃ | *q_max_* (mg·g^-1^) | 7.5442 |
|  |  | *K_L_* (L·mg^-1^) | 0.1657 |
|  |  | *R*^2^ | 0.9947 |
| Freundlich | 25℃ | *K_F_* (L·g^-1^) | 0.5798 |
|  |  | n^-1^ | 0.6697 |
|  |  | *R*^2^ | 0.9949 |
|  | 35℃ | *K_F_* (L·g^-1^) | 1.2853 |
|  |  | n^-1^ | 0.4673 |
|  |  | *R*^2^ | 0.9706 |
|  | 45℃ | *K_F_* (L·g^-1^) | 2.2493 |
|  |  | n^-1^ | 0.3146 |
|  |  | *R*^2^ | 0.9072 |

**Table S6.** Temkin and D-R isotherm model parameters of adsorption for DMP on BMS@TiO_2_.

| Model | Temperature | Parameter | Values |
| --- | --- | --- | --- |
| Temkin | 25℃ | *B* | 2.6932 |
|  |  | *K_T_* (L·mg^-1^) | 0.2808 |
|  |  | *R*^2^ | 0.9996 |
|  | 35℃ | *B* | 2.1266 |
|  |  | *K_T_* (L·mg^-1^) | 0.5891 |
|  |  | *R*^2^ | 0.9977 |
|  | 45℃ | *B* | 1.6352 |
|  |  | *K_T_* (L·mg^-1^) | 1.7279 |
|  |  | *R*^2^ | 0.9923 |
| D-R model | 25℃ | *q_max_* (mol·g^-1^) | 4.70ⅹ10^-4^ |
|  |  | *B_DR_* (mol^2^·KJ^2^) | 5.94ⅹ10^-9^ |
|  |  | *E* (KJ·mol^-1^) | 9.1747 |
|  |  | *R*^2^ | 0.9951 |
|  | 35℃ | *q_max_* (mol·g^-1^) | 2.53ⅹ10^-4^ |
|  |  | *B_DR_* (mol^2^·KJ^2^) | 4.63ⅹ10^-9^ |
|  |  | *E* (KJ·mol^-1^) | 10.3919 |
|  |  | *R*^2^ | 0.9759 |
|  | 45℃ | *q_max_* (mol·g^-1^) | 1.29ⅹ10^-4^ |
|  |  | *B_DR_* (mol^2^·KJ^2^) | 2.98ⅹ10^-9^ |
|  |  | *E* (KJ·mol^-1^) | 12.9532 |
|  |  | *R*^2^ | 0.9196 |

**Table S7.** The thermodynamic parameters of adsorption for DMP onto BMS@TiO_2_.

| T (℃) | Δ*H*° (kJ·mol^-1^) | Δ*S*° (J·mol^-1^·K^-1^) | Δ*G* (kJ·mol^-1^) |
| --- | --- | --- | --- |
| 25 | 70.40 | 343.58 | -31.94 |
| 35 |  |  | -35.67 |
| 45 |  |  | -38.80 |

**Table S8.** Kinetic model parameters of photodegradation for DMP by BMS@TiO_2_.

|  | Pseudo-first order | | Pseudo-second order | |
| --- | --- | --- | --- | --- |
| Parameter | *k*_1_ (h^-1^) | *R*^2^ | *k*_2_ (h·mg^-1^) | *R*^2^ |
| Values | 0.2281 | 0.9813 | 0.6081 | 0.9187 |

**Figure**s


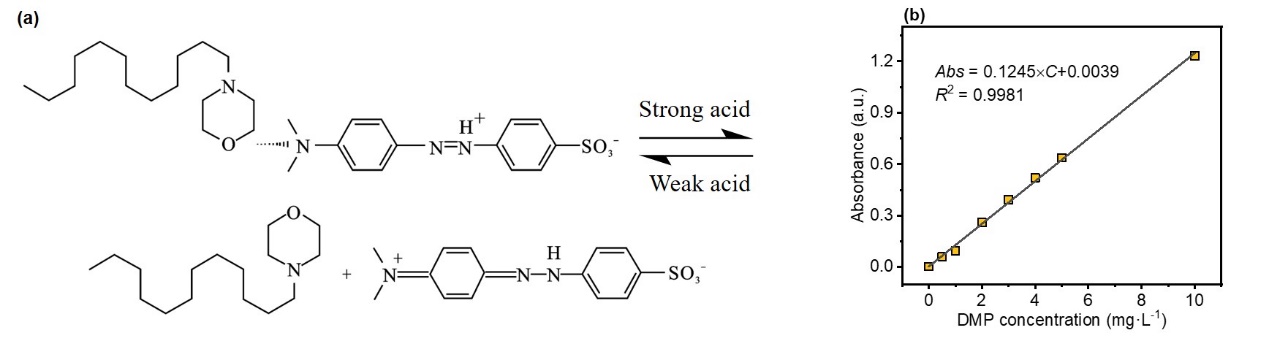


**Fig. S1.** (a) Schematic diagram of the formation and decomposition process of methyl orange and DMP complexes, and (b) DMP concentration standard curve.


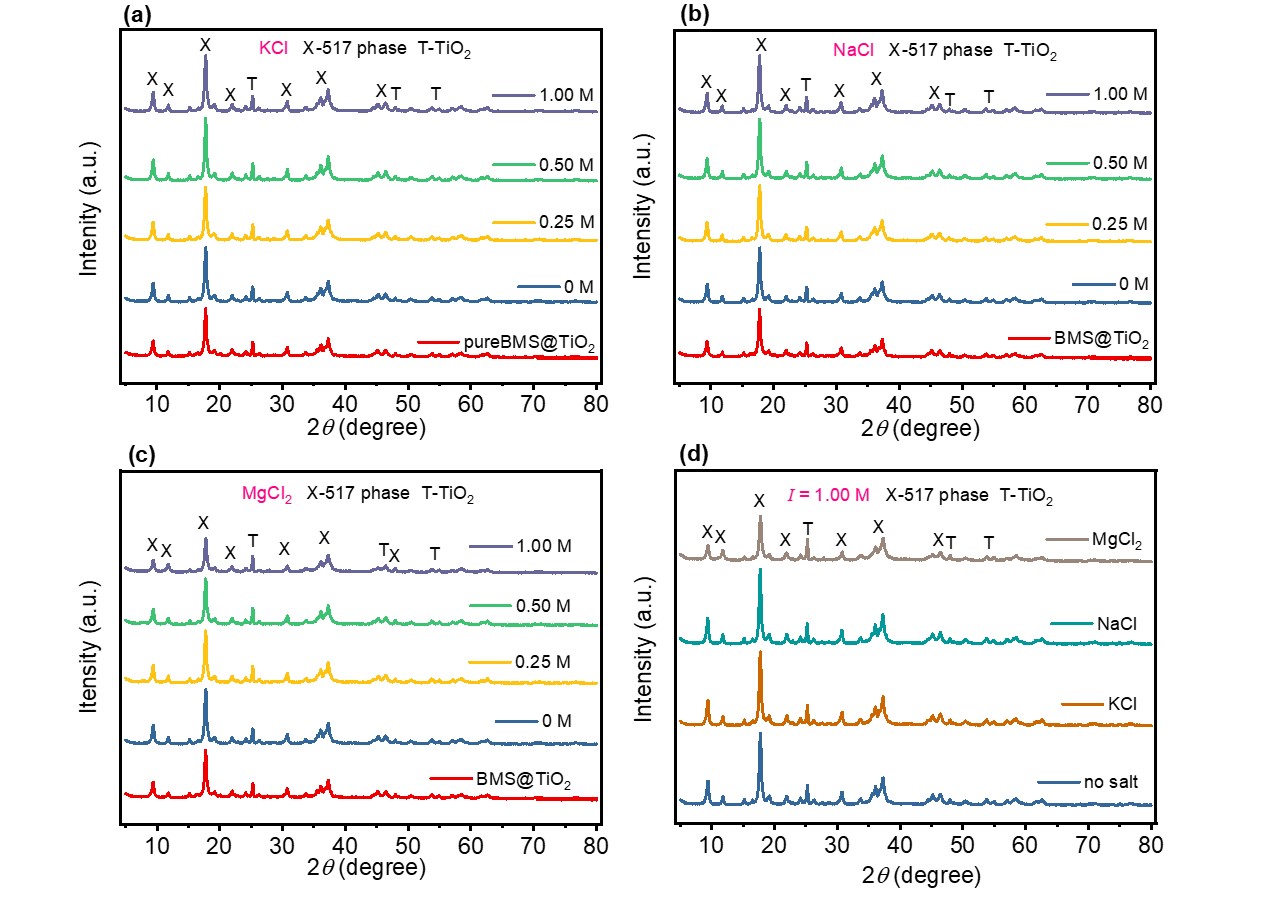


**Fig. S2.** XRD patterns of effect of co-existing salt: (a) KCl, (b) NaCl, and (c) MgCl_2_, (d) XRD patterns of 1 mol·L^-1^ ionic strength with different salts.


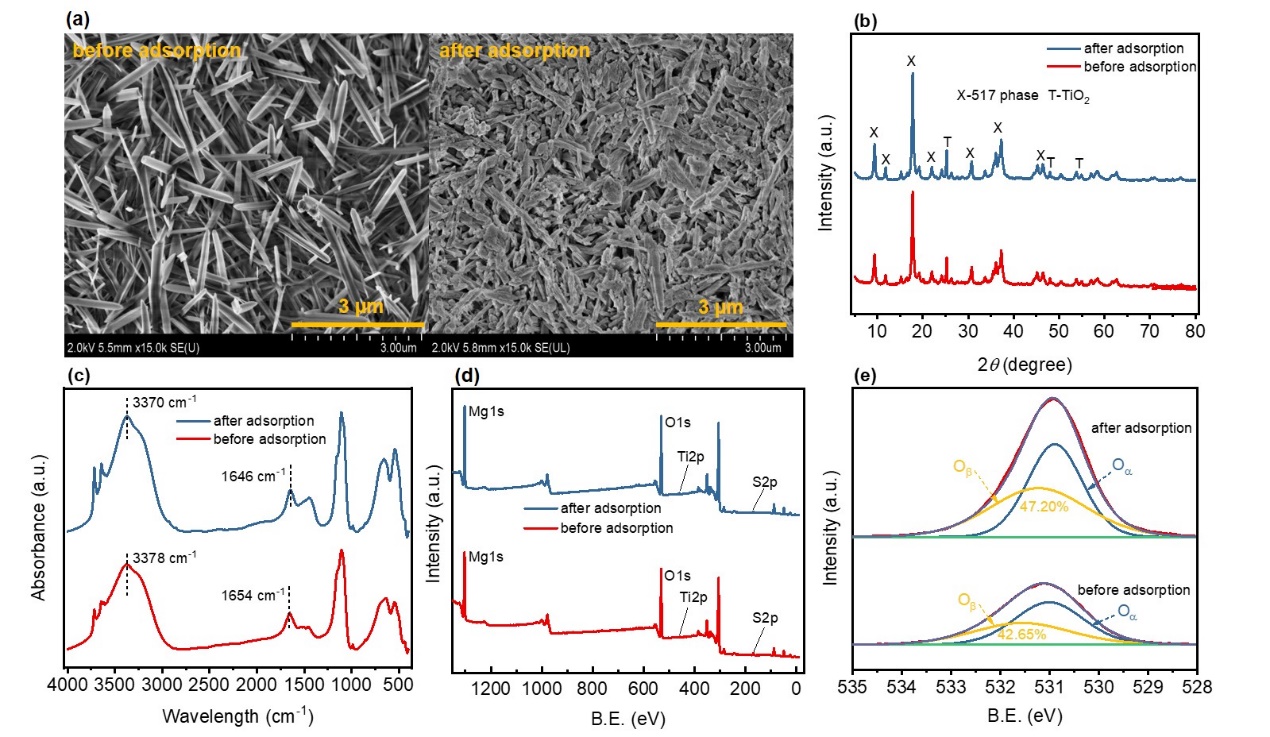


**Fig. S3.** (a) SEM images, (b) XRD patterns, (c) FTIR spectra, (d) Survey XPS spectra and (e) O1s before and after adsorption.


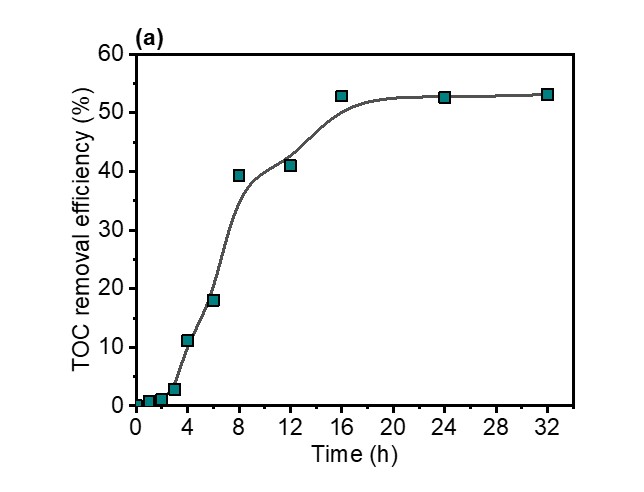


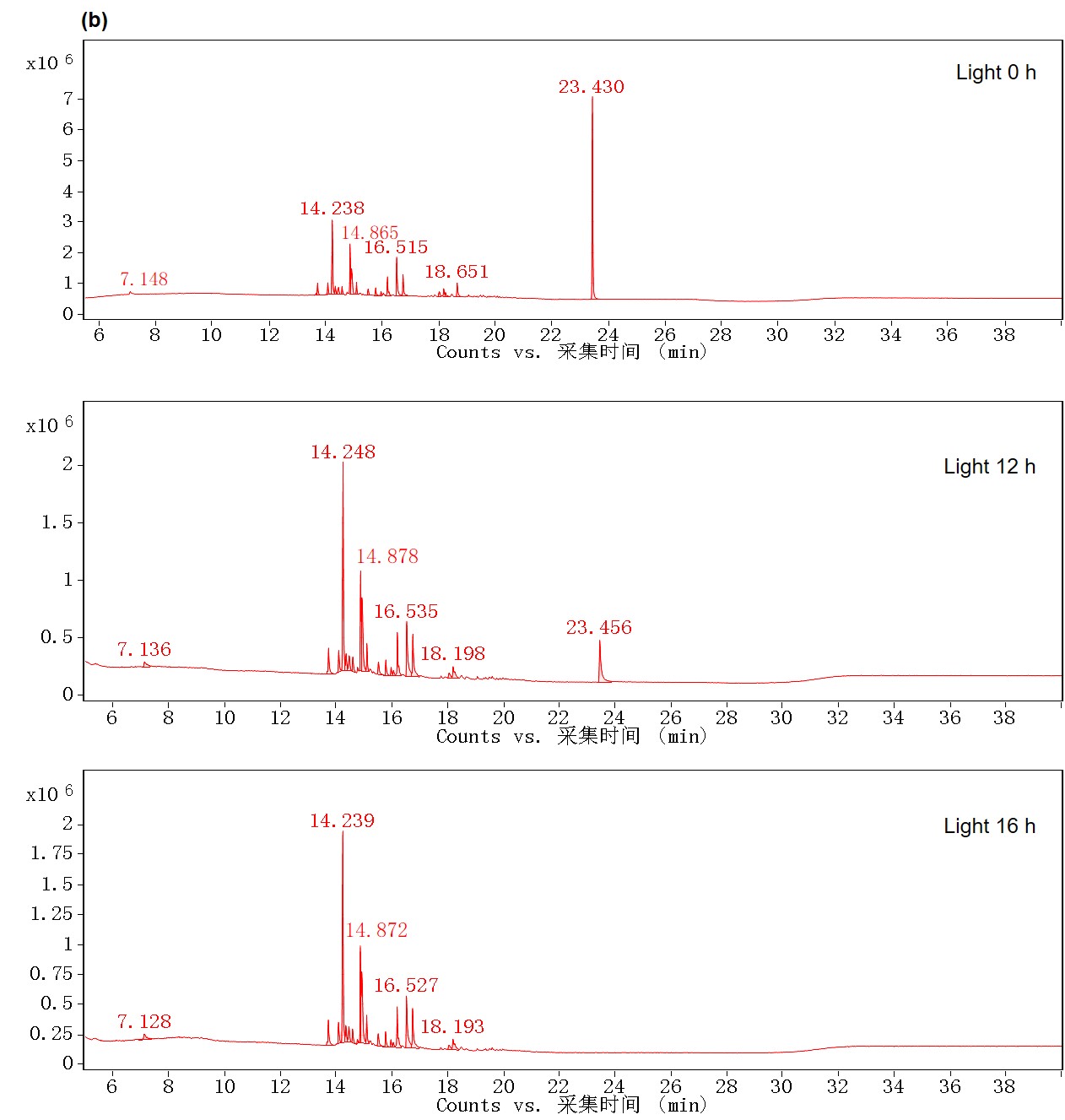


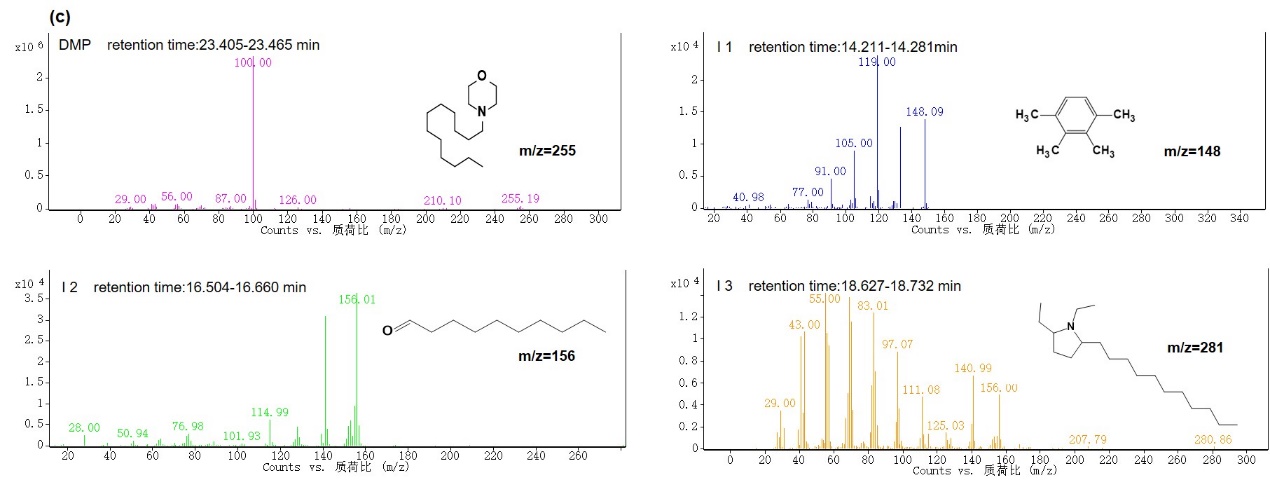


**Fig. S4.** (a) TOC removal efficiency of DMP adsorbed on BMS@TiO_2_ by photocatalytic degradation, (b) Total gas chromatogram of DMP at 0, 12, and 16 hours of photocatalytic degradation by BMS@TiO_2_, and (c) Mass spectra of main substances appeared in the gas chromatogram.

**References**

1. Gao, M.W., Li, B., Liu, J., Hu, Y.N., Cheng, H.F. Adsorption behavior and mechanism of *p*-arsanilic acid on a Fe-based metal-organic framework. *J. Colloid Interface Sci.* **629,** 616-627 (2023).
2. Vijayaraghavan, K., Palanivelu, K., Velan, M. Biosorption of copper (II) and cobalt (II) from aqueous solutions by crab shell particles. *Bioresour. Technol.* **97,** 1411-1419 (2006).
3. Ayawei, N., Ekubo, A.T., Wankasi, D., Dikio, E.D. Adsorption of Congo Red by Ni/Al-CO_3_: Equilibrium, Thermodynamic and Kinetic Studies. *Orient. J. Chem.* **31,** 1307 (2015).
4. Tseng, R.L., Wu, P.H., Wu, F.C., Juang, R.S. A convenient method to determine kinetic parameters of adsorption processes by nonlinear regression of pseudo-nth-order equation. *Chem. Eng. J.* **237,** 153-161 (2014).
5. Parimaladevi, P., Venkateswaran, V. Adsorption of cationic dyes (rhodamine B and methylene blue) from aqueous solution using treated fruit waste. J*. Appl. Technol. Environ. Sanit.* **1,** 285-293(2011).
6. Hasanzadeh, M., Simchi, A., Shahriyari Far, H. Nanoporous composites of activated carbon-metal organic frameworks for organic dye adsorption: Synthesis, adsorption mechanism and kinetics studies. *J. Ind. Eng. Chem.* **81,** 405-414 (2020).
7. Zhou, X.Y., Zhou, X. The unit problem in the thermodynamic calculation of adsorption using the Langmuir equation. *Chem. Eng. Commun.* **201,** 1459-1467 (2014).
8. Frisch, M.J. et al. *Gaussian 16 Revision A 03* (Gaussian, 2016).
9. Humphrey, W., Dalke, A., Schulten, K. VMD: Visual molecular dynamics. *J. Mol. Graphics* **14,** 33-38(1996).
10. U.S. Environmental Protection Agency. Comptox Chemicals Dashboard. https://comptox.epa.gov/dashboard/chemical/details/DTXSID4042171 (accessed January 23, 2024).

1. * Corresponding authors. *E-mail addresses*: [zhanghf@isl.ac.cn](mailto:zhanghf@isl.ac.cn) (H.F. Zhang); [wuchengyou86@163.com;](mailto:wuchengyou86@163.com;) [liuhn@isl.ac.cn](mailto:liuhn@isl.ac.cn) (H.N. Liu). [↑](#footnote-ref-1)
2. [↑](#footnote-ref-2)
3. [↑](#footnote-ref-3)
